# HIV gp120 Induces Mucus Formation in Human Bronchial Epithelial Cells through CXCR4/α7-Nicotinic Acetylcholine Receptors 

**DOI:** 10.1371/journal.pone.0077160

**Published:** 2013-10-14

**Authors:** Sravanthi Gundavarapu, Neerad C. Mishra, Shashi P. Singh, Raymond J. Langley, Ali Imran Saeed, Carol A. Feghali-Bostwick, J. Michael McIntosh, Julie Hutt, Ramakrishna Hegde, Shilpa Buch, Mohan L. Sopori

**Affiliations:** 1 Respiratory Immunology Division, Lovelace Respiratory Research Institute, Albuquerque, New Mexico, United States of America; 2 Pulmonary and Critical Care Medicine, University of New Mexico, Albuquerque, New Mexico, United States of America; 3 Division of Pulmonary, Allergy, and Critical Care Medicine, University of Pittsburgh School of Medicine, Pittsburgh, Pennsylvania, United States of America; 4 George E. Wahlen Veterans Affairs Medical Center, Salt Lake City, Utah, United States of America; 5 Departments of Psychiatry and Biology, University of Utah, Salt Lake City, Utah, United States of America; 6 The Department of Molecular and Integrative Physiology, Kansas University Medical Center, Kansas City, Kansas, United States of America; 7 Department of Pharmacology and Experimental Neuroscience, University of Nebraska Medical Center, Omaha, Nebraska, United States of America; George Mason University, United States of America

## Abstract

Lung diseases such as chronic obstructive pulmonary disease (COPD), asthma, and lung infections are major causes of morbidity and mortality among HIV-infected patients even in the era of antiretroviral therapy (ART). Many of these diseases are strongly associated with smoking and smoking is more common among HIV-infected than uninfected people; however, HIV is an independent risk factor for chronic bronchitis, COPD, and asthma. The mechanism by which HIV promotes these diseases is unclear. Excessive airway mucus formation is a characteristic of these diseases and contributes to airway obstruction and lung infections. HIV gp120 plays a critical role in several HIV-related pathologies and we investigated whether HIV gp120 promoted airway mucus formation in normal human bronchial epithelial (NHBE) cells. We found that NHBE cells expressed the HIV-coreceptor CXCR4 but not CCR5 and produced mucus in response to CXCR4-tropic gp120. The gp120-induced mucus formation was blocked by the inhibitors of CXCR4, α7-nicotinic acetylcholine receptor (α7-nAChR), and γ-aminobutyric acid (GABA)_A_R but not the antagonists of CCR5 and epithelial growth factor receptor (EGFR). These results identify two distinct pathways (α7-nAChR-GABA_A_R and EGFR) for airway mucus formation and demonstrate for the first time that HIV-gp120 induces and regulates mucus formation in the airway epithelial cells through the CXCR4-α7-nAChR-GABA_A_R pathway. Interestingly, lung sections from HIV ± ART and simian immunodeficiency virus (SIV) ± ART have significantly more mucus and gp120-immunoreactivity than control lung sections from humans and macaques, respectively. Thus, even after ART, lungs from HIV-infected patients contain significant amounts of gp120 and mucus that may contribute to the higher incidence of obstructive pulmonary diseases in this population.

## Introduction

Prior to the advent of anti-retroviral therapy (ART), pulmonary diseases were frequent complications of HIV infection [1]. Interestingly, however; while HIV-associated mortality has decreased substantially after the introduction of ART [2], lung diseases continue to remain a major cause of morbidity and mortality among HIV patients [3]. HIV-infected patients exhibit a significantly higher incidence and early onset of chronic obstructive pulmonary disease (COPD), chronic bronchitis, asthma and lung infections [4-6]. For example, it was reported that 23% of relatively young (mean age 34 years) HIV-infected smokers without a history of pulmonary infections developed COPD/emphysema as detected by computer tomography scan and lung function testing, compared to only 2% of control subjects matched for age and smoking history [4]. Although the incidence of chronic bronchitis, asthma, and COPD is much higher in smokers than never smokers, and smoking is more prevalent in HIV-infected patients, HIV may be an independent risk factor for these diseases [4,5]. The mechanism(s) by which HIV infection promotes lung disease even in the presence of ART is not clear; however, under conditions of controlled HIV viremia, the virus may persist in reservoirs leading to low levels of viral RNA and/or proteins [7]. The HIV envelop glycoprotein gp120 is associated with many HIV-related pathologies and may be present in the plasma, lymphoid tissues, and brains of HIV-infected patients and simian immunodeficiency virus (SIV)-infected monkeys before and after ART [7,8]. Moreover, lungs may harbor significant levels of the latent virus [9] and pulmonary infections may activate the latent virus [10]. 

Airway mucus overproduction is a common characteristic of lung diseases such as chronic bronchitis, COPD, and asthma. While airway mucus plays an important role in mucociliary clearance and is the first line of defense against inhaled pathogens and particulate matter [11], excessive mucus contributes to airway obstruction and pathogenesis of COPD, airway inflammation, asthma, and chronic bronchitis [12]. Excessive mucus is also an excellent milieu for bacterial growth and encourages lung infections [13]. We and others have demonstrated that airway mucus formation is strongly influenced by gamma aminobutyric acid (GABA)_A_R [14-16] and nicotinic acetylcholine receptors (nAChRs) (17) in the airway epithelial cells, and reciprocally, nAChR antagonists suppress allergen and cigarette smoke (CS)/nicotine-induced airway mucus formation both *in vitro* and *in vivo* [16]. Moreover, we have identified GABA_A_Rα2 as the GABA_A_R subtype that increases in nicotine/IL-13-treated normal human bronchial epithelial (NHBE) cells and allergen and/or CS treated mouse lungs [17]. In this communication we present evidence that HIV gp120 induces mucus formation in NHBE cells through the HIV co-receptor CXCR4 using the α7-nAChR-GABA_A_Rα2 pathway but not the epithelial growth factor receptor (EGFR) pathway. Moreover, even after ART, autopsied lungs tissues from HIV- and SIV-infected humans and monkeys, respectively, show the presence of gp120, mucus, and GABA_A_Rα2 expression. 

## Materials and Methods

### Cell Cultures

NHBE cells and culture media were purchased from Lonza (Basel, Switzerland). The cells were grown to differentiate at the air-liquid interphase (ALI) using standard procedures [17]. Alternatively, pre-differentiated NHBE cells (EpiAirway™ Tissue Model) were purchased from Mattek (Ashland, MA). 

### SIV infection

Two to three year-old Indian rhesus macaques (*Macaca mulatta*) were purchased from the Caribbean Research Primate Center and housed individually in an AAALAC-approved animal facility at the University of Kansas Medical Center. The monkeys were exposed, daily, to 12-hour light-dark cycles and given laboratory chow and water *ad libitum* along with daily snacks. All cages were equipped with environmental enrichments. The animals were tested for tuberculosis, herpes B virus and simian retrovirus and found negative in all these tests. All animal protocols were approved by the IACUC. SIVmacR71/17E was originally prepared from pooled brain homogenates from macaques infected with R17 and R17E [18,19]. Virus stock was prepared from Con A-activated peripheral blood mononuclear cells depleted of CD8^+^ T cells, and assayed for infectivity in CEMx174 cells and by plaque assay in GHOST Hi5 cells as described [20]. Animals were inoculated intravenously with approximately 10^4^ PFU of the virus. Where indicated, ART (tenofovir and emtrictabine) was given daily orally. SIV-infected animals remained “healthy” and did not show signs of suffering – wasting, poor appetite, agitation, sudden change in behavior, etc. 

### Lung tissue collection from macaques

Macaques were maintained in class 100 clean-air rooms, and at the time of sacrifice, the animals did not show any overt signs of lung infection. Animals were anesthetized with an intramuscular injection of ketamine (3mg/kg) and medetomidine (0.15mg/kg) and exsanguinated from the descending aorta. Tissue samples were fixed in 10% formalin for histopathological analysis. All animal protocols were approved by the local animal care committee (IACUC) at the University of Kansas in accordance with the *Guide for the Care and Use of Laboratory Animals*. 

### Human Lung Tissues and Sections

Frozen and paraffin-embedded sections from HIV-infected, HIV-infected on HAART, and HIV-negative- intravenous drug users (IVDU) were obtained from the Manhattan HIV Brain Bank (New York, NY). The IVDU in this study were mainly heroin and/or cocaine abusers [21]. We only examined tissues sections from those patients where the death was not attributed to lung disease and there were no overt histological signs of pneumonia. Although the history of smoking was not available, lung histology did not show detectable accumulation of cigarette smoke residue, suggesting the absence of recent smoking. Normal (control) lung tissues were collected for emphysema studies at the Division of Pulmonary, Allergy, and Critical Care Medicine, University of Pittsburg. The Institutional Review Board of the University of Pittsburgh approved obtaining lung tissues from transplant patients. Participants provided written informed consent.

### Mucus staining and Immunohistochemistry

NHBE cells at ALI were fixed in 10% neutral buffered formalin and embedded in paraffin; 5-µm-thick sections were stained for mucus with Alcian-blue/periodic acid Schiff (AB-PAS) [22,23]. Briefly, after deparaffinization, the slides were stained with AB solution at pH 2.5 for 30 min, washed, and treated with 1% periodic acid for 10 min. The slides were stained with Schiff’s reagent for 10 min, rinsed 3x with sodium metabisulfite, washed, dehydrated, mounted, and examined microscopically at indicated magnification. Human and macaque lung tissue sections were also stained for mucus following the same procedure as mentioned above. Quantification of mucus positive cells per millimeter of basal lamina was performed using the VisioMorph system (Visiopham, Horsholm, Denmark).

Immunohistochemical staining was performed according to the protocol previously described [23]. Mouse Monoclonal anti-MUC5AC (45M1; Thermo Scientific, Lafayette, CO) and rabbit polyclonal anti-GABA_A_Rα2 (Sigma, St. Louis, MO) antibodies were used at pre-optimized concentrations of 1:100 and 1:50 dilutions, respectively. The tissue sections were treated with the primary antibodies overnight at 4°C, followed by incubation with biotinylated secondary antibody (VECTASTAIN^®^ Elite ABC kit, Vector Laboratories, Burlingame, CA). Binding was visualized using an avidin-biotinylated enzyme complex (VECTASTAIN^®^ Elite ABC kit) with 3, 3’-diaminobenzidine (DAB) as substrate.

### Cell treatments

Unless indicated otherwise, NHBE cells were treated with 100 ng/ml of HIV-1 gp120_LAV_ (Ray Biotech Inc, Norcross, GA), and 100 ng/ml of gp120 96ZM651 (gp120_ZM_) and gp120_MN_ (Dr. Madhavan Nair; Florida International University, Miami, FL). The inhibitors of nAChRs, EGFR, CXCR4, and CCR5 were purchased from Sigma (St. Louis). The inhibitors were used at the following concentrations: α7-nAChR inhibitors MLA (1 μM), CXCR4 inhibitor AMD3100 (10 μM), EGFR inhibitor AG1478 (5 μM), and CCR5 inhibitor Maraviroc (10 μM), and added to the cell cultures 2 h prior to the addition of gp120 or EGF. The highly specific conotoxin peptide inhibitor for α7-nAChRs ArIB was prepared as described previously [24] and used at 500 nM [16].

### RT-PCR and qPCR

Total RNA from human and macaque lungs and NHBE cells was isolated using TRI-Reagent (Molecular Research Center, Inc., Cincinnati, OH). qPCR was performed using Step One plus Detection System (Applied Biosystems, Foster City, CA) and TaqMan One-Step RT-PCR kit containing AmpliTaq Gold^®^ DNA polymerase. Specific primers and probes for MUC5AC and GABA_A_Rα2 were obtained from Applied Biosystems. Fold changes in qPCR expression were calculated by the 2^(-ΔΔCT)^ method [25]. 

### Statistical analysis

All data were analyzed using Graph-Pad Prism software 5.03 (Graph-Pad Software Inc., San Diego, CA). One-way ANOVA was used to compare mean between the groups using Turkey post-hoc test that compares all groups at 95% confidence intervals. Results are presented as the means + SEM. The differences with p value of <0.05 were considered statistically significant. *P ≤0.05; **P ≤0.01, ***P ≤0.001.

## Results

### HIV gp120 promotes mucus formation in NHBE cells

**Figure 1:**
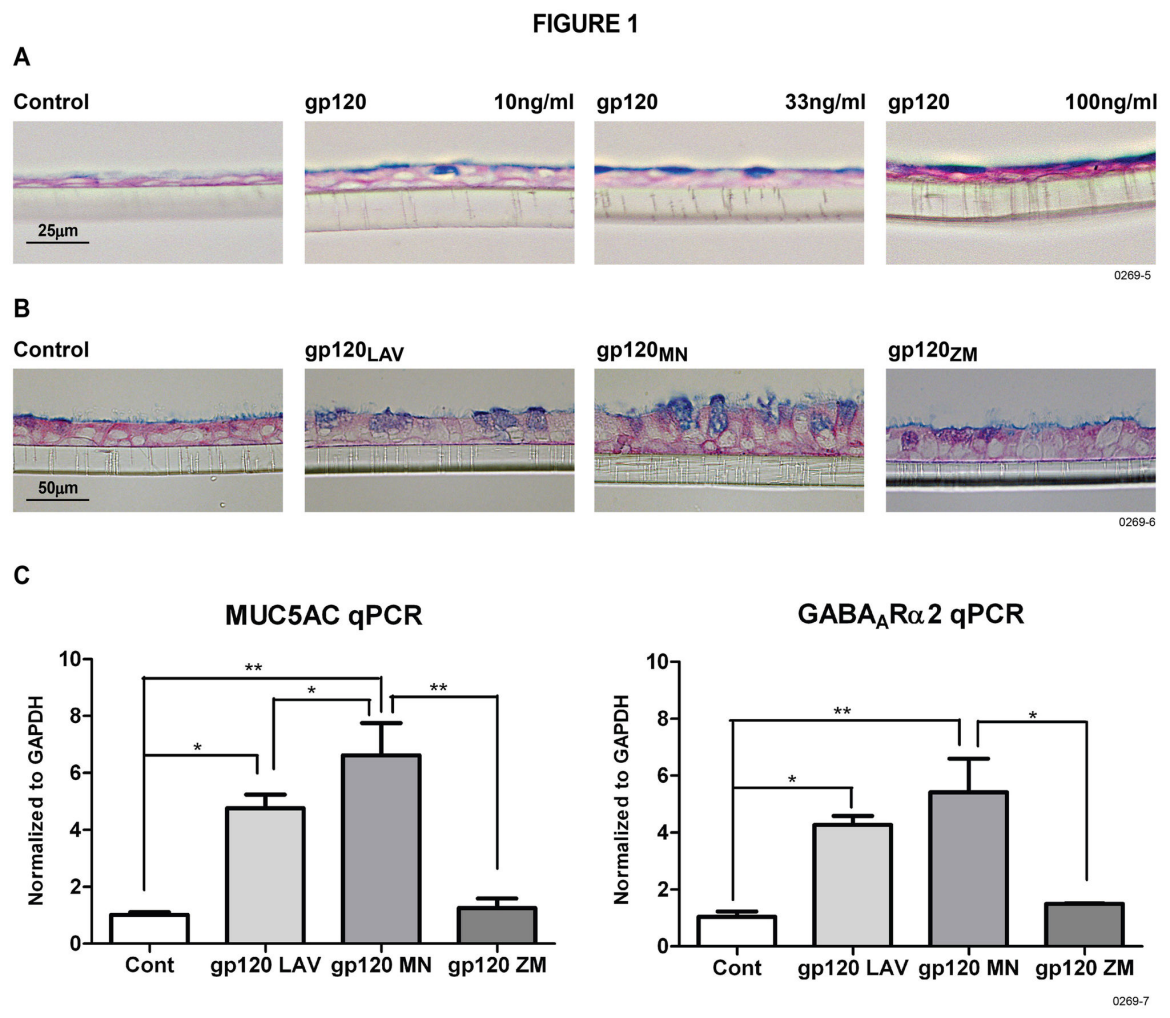
HIV gp120 induces mucus formation and increases MUC5AC and GABA_A_Rα2 expression in NHBE cells. (**A**): NHBE cells were treated with 10, 33, and 100 ng/ml of gp120_LAV_ for 48 hr and the mucus in the inserts was visualized by AB-PAS staining. (**B**): NHBE cells were treated with 100 ng/ml of gp120_LAV_, gp120_MN_, or gp120_ZM_ and examined for mucus formation by AB-PAS staining. (**C**): The expression of MUC5AC and GABA_A_Rα2 by qPCR. Each experiment was repeated at least three times. Error bars on MUC5AC and GABA_A_Rα2 qPCR analysis represent mean ± SEM from three inserts.

HIV may be an independent risk factor for COPD, asthma, and bronchitis [5,26], and mucus formation in the lung is a characteristic of these diseases (13). HIV gp120 has been implicated in number of HIV-associated pathologies [27,28]; therefore, we ascertained whether HIV gp120 in realistic concentrations triggered mucus formation in NHBE cells grown on ALI [16]. We used a concentration of 10-100 ng/ml (8.3 x 10^-11^ to 8.3 X 10^-10^ M) of X4-gp120 (gp120_LAV_) in NHBE cell cultures. The rationale for using these concentrations was that many HIV patients on HAART have gp120 levels in plasma/tissues that are within this concentration range [8] and some may even exceed 700 ng/ml [7,29]. Similarly, macaques infected with chimeric simian-human immunodeficiency virus (SHIV) exhibit gp120 concentrations ranging from 183-562 ng/ml in lymphoid mononuclear cell lysates [30]. Figure 1A shows that as little as 10 ng/ml of gp120 (83 pM) induces detectable mucus levels in NHBE cells. Optimal mucus production was noted at a gp120 concentration of 33-100 ng/ml and, therefore, unless mentioned otherwise, 100 ng/ml of gp120_LAV_ (gp120) was used in NHBE cell cultures. 

### HIV gp120 induces mucus through CXCR4 receptors on NHBE cells

**Figure 2:**
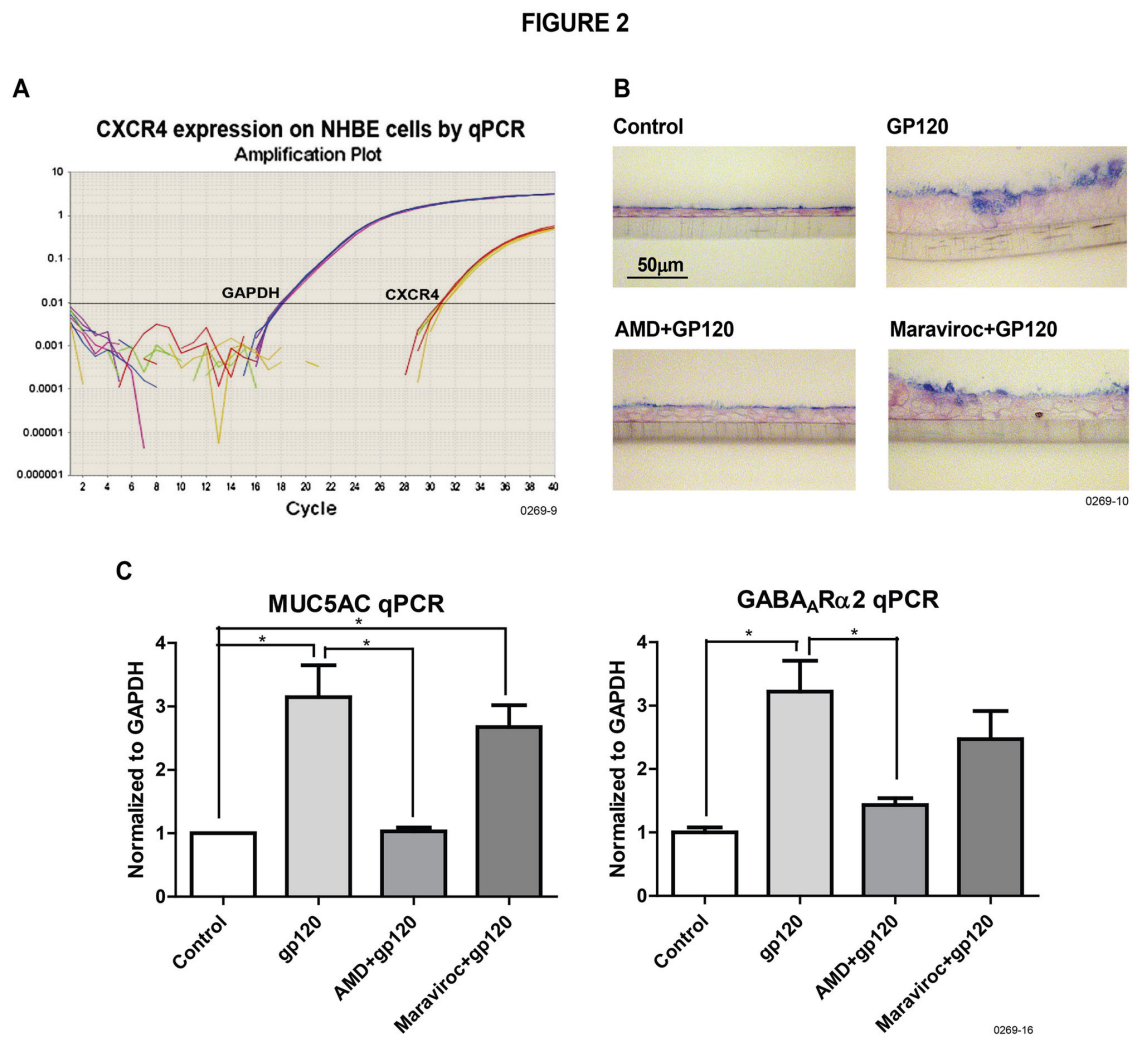
NHBE cells express CXCR4 but not CCR5 and HIV gp120 induces mucus through CXCR4. (**A**): NHBE cell RNA was analyzed by qPCR for the expression of CXCR4 and CCR5 up to 40 cycles of amplification. (**B**): gp120_LAV_-induced mucus formation was determined in the presence or absence of CXCR4 inhibitor AMD3100 and CCR5 inhibitor maraviroc. (**C**): gp120-induced mRNA expression of MUC5AC and GABA_A_Rα2 was determined in the presence and absence of AMD3100 and maraviroc by qPCR analysis. Each experiment was repeated at least three times; bars represent mean ± SEM.

CXCR4 and CCR5 are the major coreceptors for HIV gp120; therefore, we determined whether NHBE cells: (a) responded differentially to CXCR4 (X4)- or CCR5 (R5)-tropic HIV-gp120, and (b) expressed CXCR4 and/or CCR5 HIV coreceptors. We used gp120 variants, including gp120_LAV_ (X4-tropic), gp120_MN_ (R5/X4 dual-tropic), and gp120_ZM_ (R5-tropic). Compared to the R5-tropic gp120_ZM_, X4-tropic gp120_LAV_ and R5/X4-dualtropic gp120_MN_ strongly induced mucus formation and mucous cell metaplasia in NHBE cells (Figure 1B). Similarly, as detected by qPCR, the expression of the major inducible mucin in the airway mucus, MUC5AC (Figure 1C, left panel) - and GABA_A_Rα2 (Figure 1C, right panel) were upregulated more strongly by X4- and X4/R5- than R5-tropic gp120, suggesting gp120-X4 is a strong stimulator of mucus formation in NHBE cells. Because even the R5-tropic gp120_ZM_ modestly increased mucus formation in NHBE cells, we determined the expression of CXCR4 and/or CCR5 in NHBE cells by qPCR. Results presented in Figure 2A indicate that while NHBE cells express CXCR4, expression of CCR5 was not detected up to 40 cycles of qPCR. The induction of mucus by gp120_LAV_ (Figure 2B), and gp120_MN_ and gp120_ZM_ (not shown) was blocked by pretreatment of NHBE cells with CXCR4 antagonist AMD3100, but not by the CCR5 antagonist maraviroc, and qPCR analysis indicated that gp120-induced expression of MUC5AC (Figure 2C, left panel) and GABA_A_Rα2 (Figure 2C, right panel) was suppressed by AMD3100 but not by maraviroc. Thus, in NHBE cells gp120 promotes mucus formation through CXCR4. 

### HIV gp120 induces mucus formation via the α7-nAChR/GABA_A_Rα2 pathway in NHBE cells

**Figure 3:**
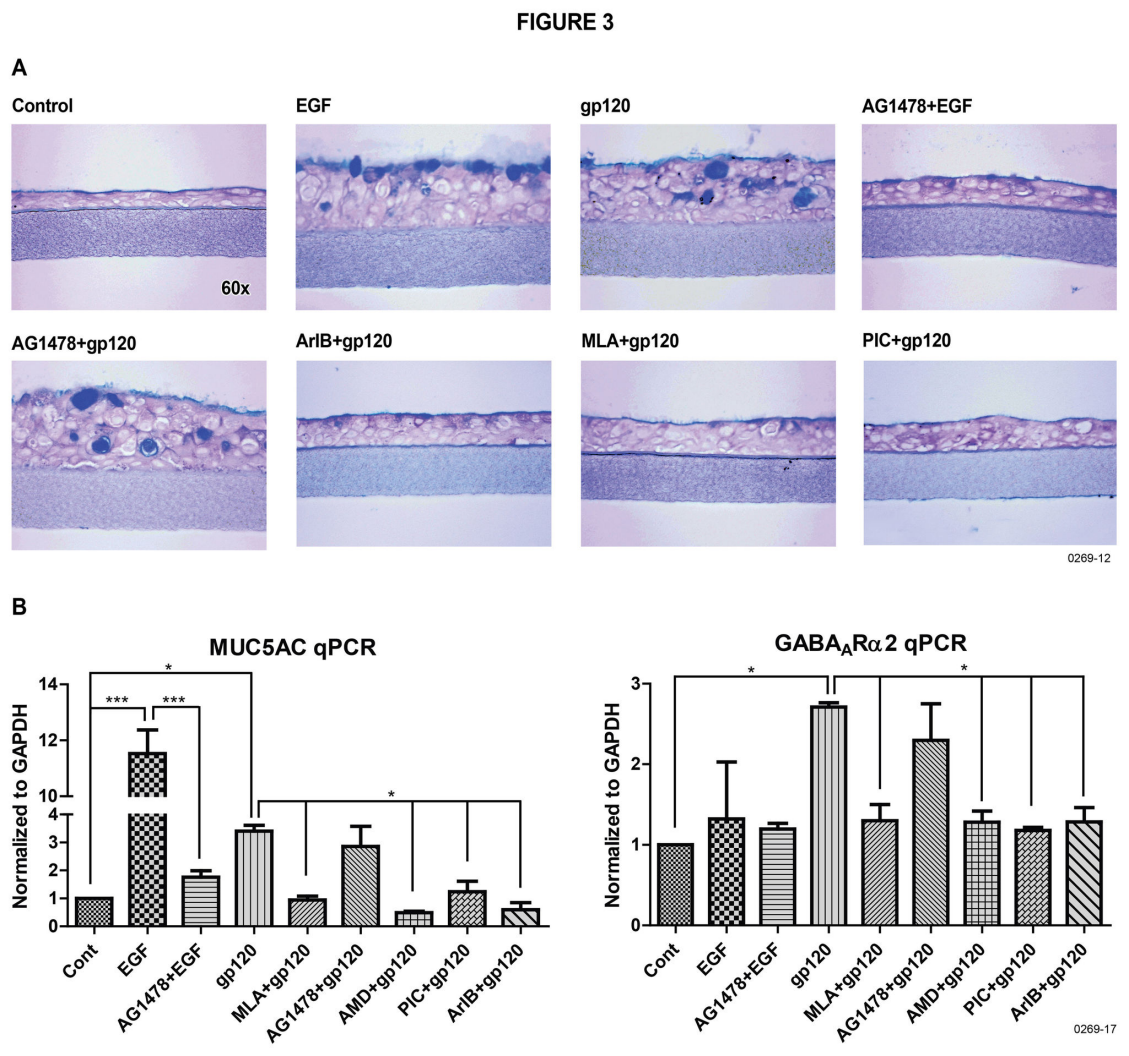
HIV gp120 induces mucus formation through α7-nAChRs in NHBE cells. (**A**): NHBE cells were cultured with EGF or gp120_LAV_ as described in Materials and Methods. Where indicated, EGFR inhibitor (AG1478) or α7-nAChR inhibitors (MLA or ArIB) and GABA inhibitor picrotoxin (PIC) were added 2 h before the addition of gp120/EGF. Cells were stained for mucus (AB-PAS). (**B**): Expression of MUC5AC and GABA_A_Rα2 mRNA was determined by qPCR analysis. The results represent three independent experiments and error bars are mean ± SEM from three inserts.

We have demonstrated that nicotine *per se* stimulates mucus production in NHBE cells through the α7-nAChRs-GABA_A_Rα2 pathway [16]. Mucus formation in airway epithelial cells is also regulated by EGFR [31]. To ascertain whether the induction of mucus in NHBE cells by gp120 uses α7-nAChR and/or EGFR pathways, prior to addition of gp120, NHBE cells were incubated with α7-nChR-specific antagonists methyllycaconitine (MLA) or the conotoxin peptide ArIB[V11L, V16D] (ArlB) [16,32], or the EGFR antagonist AG1478 at predetermined optimal concentrations. As seen in Figure 3A, while AG1478 inhibited EGF stimulated mucus, it had no detectable effect on gp120-induced mucus formation. On the other hand, α7-nAChR inhibitors (MLA and ArIB) suppressed the mucus formation in NHBE cells in response to gp120. Thus HIV gp120 uses the α7-nAChR but not the EGFR pathway to induce mucus formation in NHBE cells. These results were further supported by the qPCR data showing that α7-nAChR inhibitors inhibited the expression of gp120-induced MUC5AC (Figure 3B, left panel) and GABA_A_Rα2 (Figure 3B, right panel) in NHBE cells. These results suggest that CXCR4 communicates with α7-nAChRs to regulate HIV-gp120-mediated mucus production in NHBE cells.

GABA_A_Rs have been shown to be critical in IL-13/nicotine-induced mucus formation in NHBE cells [15] and the expression of GABA_A_R subtype GABA_A_Rα2 is upregulated by IL-13 and allergens *in vitro* and *in vivo* respectively [17]. To ascertain whether gp120-induced mucus formation required GABA_A_Rs, we used the GABA_A_R inhibitor picrotoxin to block the activation of GABA_A_Rs. Pretreatment of NHBE cells with picrotoxin blocked the gp120-induced mucus formation (Figure 3A) as well as the mRNA expression of MUC5AC (Figure 3B, left panel) and GABA_A_Rα2 (Figure 3B, right panel). 

### HIV and SIV infections induce airway mucus formation

**Figure 4:**
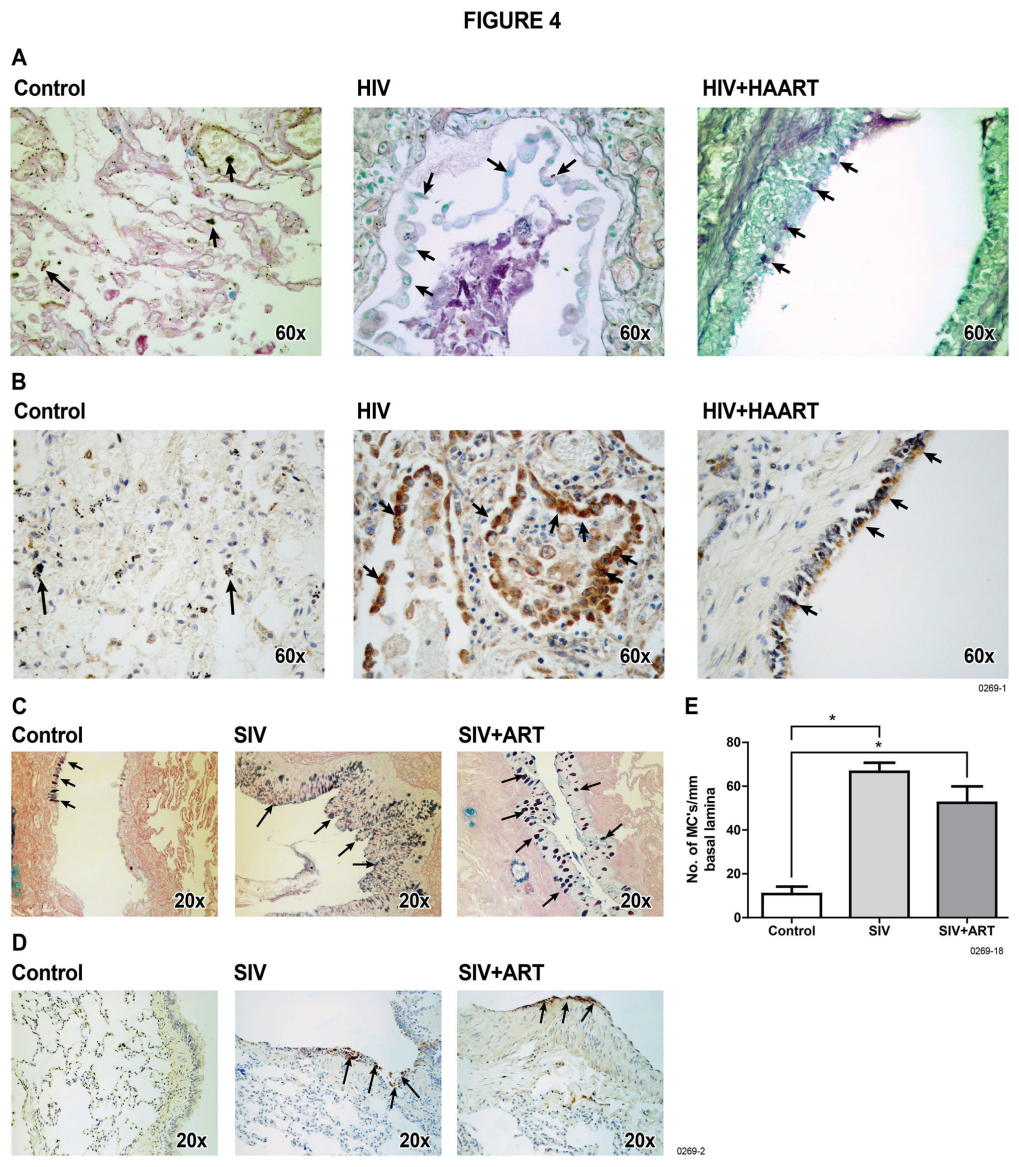
HIV and SIV infections increase mucus and GABA_A_Rα2 expression in the lung. Lung sections from control, HIV-infected, and HIV + HAART patients were (**A**): stained for AB-PAS for mucus and (**B**): stained for GABA_A_Rα2 by IHC. Similarly, lung sections from control, SIV-infected, and SIV + ART monkeys were (**C**): stained with AB-PAS for mucus and (**D**): IHC for GABA_A_Rα2. (**E**): Mucus containing cells (MC’s)/mm basal lamina were enumerated microscopically in lung sections from control, SIV-infected, and SIV + ART lung sections. Error bars in (**E**) represent data from at least 5 different lung sections from each group.

Mucus hypersecretion plays an important role in the pathogenesis of COPD, asthma, and chronic bronchitis [12,33]. To ascertain whether HIV and SIV infections are associated with mucus production in the lung, we examined autopsied lung tissues from control, HIV-infected, and HIV-infected + HAART patients. These lung sections were examined for mucus by AB-PAS staining (Figure 4A) and for GABA_A_Rα2 expression by immunohistochemistry (IHC) (Figure 4B). The results indicate that control human lungs (n = 7) have very low level of airway mucus; however, lungs from HIV-infected without HAART (n = 2) and HIV-infected + HAART (n = 6) have significantly higher levels of mucus and GABA_A_Rα2. 

Many HIV patients die from lung diseases, including lung infections. Although, we selected the autopsied tissues only from the individuals where the death was not attributed to lung diseases and histopathology did not suggest overt pneumonia or recent smoking; nonetheless, it was possible that mucus formation in HIV-infected patients resulted from lung infections. Therefore, we obtained lung tissues from uninfected (n = 2) and SIV-infected rhesus macaques without ART (n=3) and with ART treatment (n=4). Following SIV infection, these animals were held in sterile facilities and at the time of sacrifice, the monkeys were relatively healthy. Some of these monkeys were treated with ART, comprising of tenofovir and emtrictabine at 35 mg/kg each, for 28 weeks. The treatment suppressed the viremia by >99.5% (viral RNA titers before ART and after ART were 1,723,123 ± 974,510 and 7,555 ± 3520, respectively). As in HIV-negative humans, control macaque lungs had relatively little mucus; however, mucus formation was strongly increased after SIV infection and ART only modestly affected SIV-associated mucus formation in the lung (Figure 4C). Similar results were obtained when the lungs were scored for GABA_A_Rα2 expression by IHC (Figure 4D) and the number of goblet cells (MCs)/mm basal lamina microscopically (Figure 4E). Together these results suggest that both HIV and SIV infections promote mucus formation in the lung and HAART/ART does not block this response appreciably. 

### Lungs from HIV/SIV-infected and ART-treated humans and monkeys exhibit gp120 immunoreactivity

**Figure 5:**
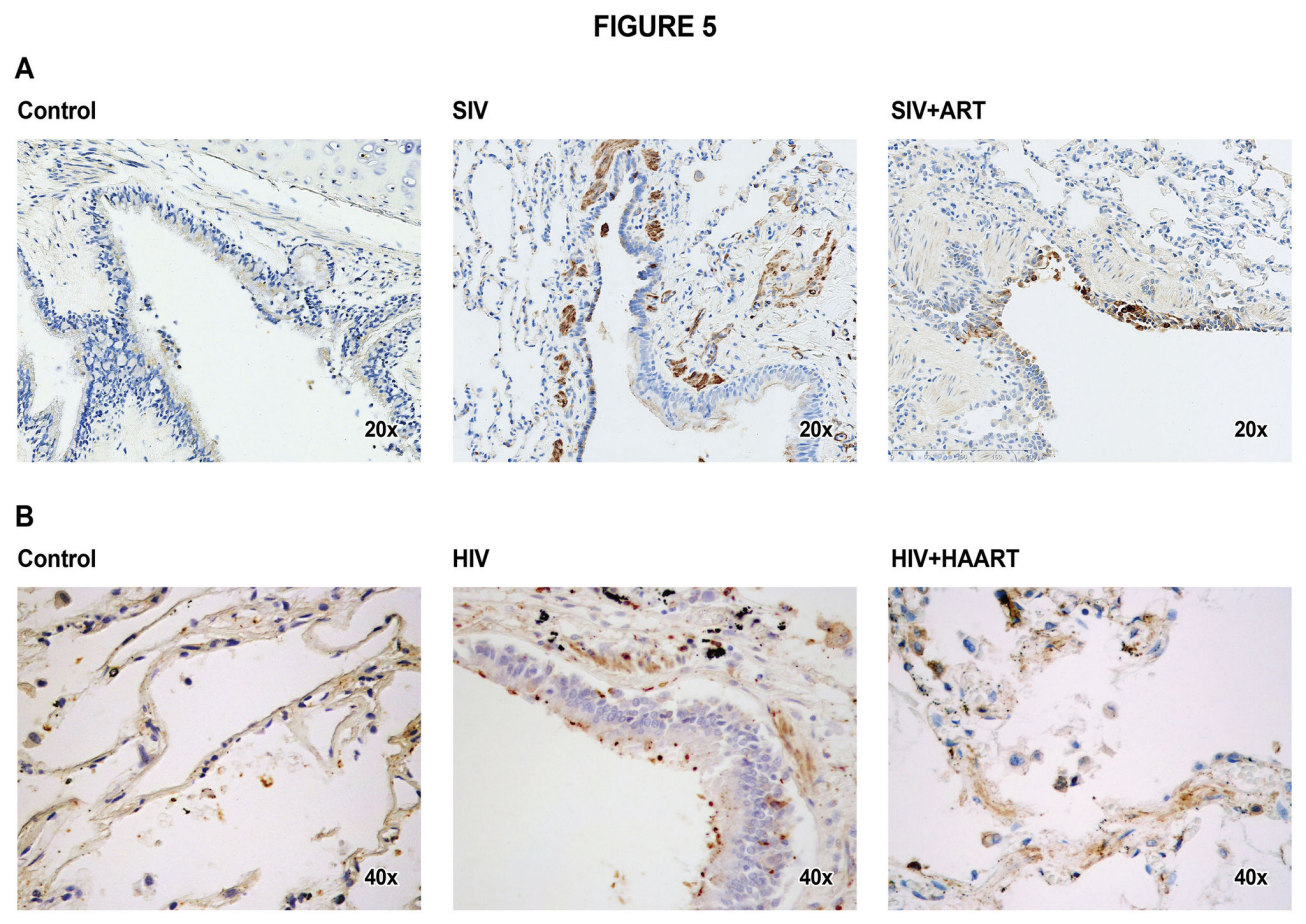
SIV- and HIV-infected lungs exhibit gp120 immunoreactivity even after ART. Lung sections from control, SIV, and SIV + ART (A), and control, HIV-infected, and HIV + HAART (B) were stained for gp120 immunoreactivity as described in Materials and Methods.

If the viral components such as gp120 were to promote airway mucus formation, it should be present in HIV/SIV-infected lungs even after ART. It is clear from IHC data that gp120 immunoreactivity is present in SIV-infected and SIV + ART macaque lungs (Figure 5A), even though ART had reduced the viremia by more than 99.5% in these animals. The gp120 immunoreactivity was widely scattered in these lungs but, in SIV + ART animals, gp120 immunoreactivity was localized mainly to macrophage-like cells in the lung. Interestingly, while the SIV-infected lungs without ART had small fibrotic areas, these fibrotic areas were essentially absent in SIV + ART animals, indicating ART suppressed the SIV-induced fibrotic response in the lung. 

HIV-gp120 immunoreactivity was also seen in the lungs of HIV patients with and without HAART (Figure 5B); however, it was difficult to determine whether HAART had any significant effect on gp120 or mucus levels in the lung. Together, these results suggest that significant amounts of gp120 are present in HIV/SIV-infected lungs even after HAART/ART.

## Discussion

Increasing evidence suggests that even after ART, mucus-associated lung diseases such as COPD, asthma, and chronic bronchitis are more common among HIV-infected than HIV-negative subjects matched for age and smoking history [4-6]. HIV-gp120 is associated with a number of HIV-related pathologies, and gp120 interacts with multiple non-infected cell types and impairs their function, including T cells [34], dendritic cells [35] and neurons [36]. In this communication, we present evidence that gp120 induces mucus formation and mucous cell metaplasia/hyperplasia in NHBE cells. 

Airway mucus has been shown to be regulated by EGFRs [36] and GABA_A_Rs [15]. We have demonstrated that IL-13/nicotine-induced mucus formation in NHBE cells is linked to GABA_A_R through α7-nAChRs [17]. Herein we have shown that while the GABA_A_R antagonist picrotoxin blocked gp120-induced mucus formation and MUC5AC expression, it failed to inhibit the EGFR-induced mucus formation in NHBE cells. Thus, GABA_A_R and EGFR are distinct pathways for mucus production in NHBE cells. Moreover, specific blockers of α7-nAChRs but not EGFR inhibited the gp120-induced mucus formation, suggesting that HIV gp120 uses the α7-nAChR-GABA_A_Rα2 pathway but not EGFR pathway to promote mucus formation in NHBE cells. This observation has implication for HIV-infected smokers, because a large number of HIV-infected patients are tobacco smokers [37] and both CS/nicotine and HIV use the α7-nAChR-GABA_A_Rα2 pathway to induce mucus production in the lung. 

HIV-gp120 interacts with several HIV coreceptors, including CXCR4 and CCR5 [38]. Therefore, to ascertain how gp120 interacts with NHBE cells, we determined the status of CCR5 and CXCR4 on NHBE cells. Analysis by qPCR indicated that while the expression of CCR5 was undetectable, CXCR4 was present in NHBE cells. Reciprocally, X4- and X4/R5 dual tropic gp120 were much stronger stimuli for mucus formation in NHBE cells than R5-tropic gp120. Although, R5-tropic gp120 was not completely devoid of mucus-inducing capability, only CXCR4-specific (AMD3100) but not CCR5-specific (maraviroc) inhibitors blocked the gp120-induced mucus formation in NHBE cells. This suggested that the modest mucus-stimulating ability of R5-tropic gp120 resulted from its cross-reaction with CXCR4. 

Interestingly, HIV-1 gp120 was shown to compete with α-bungarotoxin binding in neuronal cells, indicating that gp120 may bind nAChRs in neuronal cells [39]; however, we have no evidence that gp120 binds directly to nAChRs in NHBE cells. Inhibitors of CXCR4 and α7-nAChRs suppressed gp120-induced mucus formation in NHBE cells, but the CXCR4 inhibitor did not block nicotine-induced mucus production (not shown), suggesting that α7-nAChRs are downstream of CXCR4 in the gp120-induced mucus formation in NHBE cells. More recently, binding of gp120 to CXCR4 was shown to upregulate the expression nAChRs in neuronal cells [40]. Together these results suggest that CXCR4 communicates with nAChRs in regulating biological functions, including mucus production. Moreover, high numbers of CXCR4-positive cells were found in the lungs of HIV patients even after ART [41]. Although the contribution of bronchial epithelial to the CXCR4-positive cell population in the lung in these patients is not known, ART may not completely suppress the proliferation of CXCR4-positive cells in the lung.

In order to establish the in vivo significance of the finding that HIV-gp120 stimulates mucus production in NHBE cells, we determined whether the lungs from HIV-infected subjects contained mucus and, if so, whether it involved the nAChR-GABA_A_Rα2 pathway. Indeed, the lung samples from HIV-infected subjects were strongly positive for mucus and expressed GABA_A_Rα2; HAART did not significantly decrease this response. On the other hand, lung samples from controls subjects had very little mucus and essentially lacked GABA_A_Rα2 expression. Therefore it was likely that activation of the α7-nAChR-GABA_A_Rα2 pathway in HIV-infected lungs led to mucus formation; however, it was unclear whether the mucus in HIV-infected lungs resulted from (a) secondary bacterial/fungal infections, (b) cigarette smoking, and/or (c) drug abuse among the HIV-infected patients. We selected samples where death was not attributed to lung disease; unfortunately the smoking history of the tissue donors was not known. Nonetheless, we selected the lungs that did not show smoke residues in the lung indicating that, at least in months prior to death, the donors did not smoke. Also, many of the HIV-infected lung samples were from patients who were drug abusers (primarily morphine); however, we were able to show that lung samples from HIV-negative drug abusers did not have significant mucus or GABA_A_Rα2 expression in the lung. Thus, drug abuse *per se* does not lead to excessive mucus formation in the lung; however, we were unable to ascertain the contribution of unknown non-HIV lung infections in the mucus response in HIV-infected subjects. Therefore, we decided to examine the lung tissues from SIV-infected monkeys. These animals were held in highly clean environment and were relatively healthy at the time of sacrifice. Some of these monkeys were treated with ART which suppressed their viremia by >99.5%. As with HIV-infected humans, lung samples from SIV-infected monkeys contained more mucus and GABA_A_Rα2 than control animals, and ART only modestly reduced the mucus formation. Interestingly, SIV infection without ART led to increased collagen content in the lung, indicating that in the absence of ART, SIV infection may promote lung fibrosis – a sign of tissue damage. This response was significantly attenuated in SIV + ART animals, suggesting that ART may protect/delay SIV-induced lung injury and fibrosis. Lung fibrosis is also associated with COPD/emphysema; however, because lung tissues from humans and monkeys were not properly inflated before fixation, we were unable to use morphometric analysis to determine whether HIV/SIV promoted COPD/emphysema. Overall, the results clearly suggest that HIV/SIV infection promotes mucus formation possibly through the α7-nAChR-GABA_A_Rα2 pathway.

The reason(s) for the presence of gp120 in the lungs after ART is unclear. It is possible that ART does not completely eliminate viral replication [9] from all HIV/SIV-infected lungs and/or the patients are developing resistance to ART therapy [42,43]. Interestingly, the bronchoalveolar lavage T cells from HIV patients with lung infections secrete gp120, suggesting that the lung environment may facilitate the production and secretion of gp120 [44]. It is also possible that some cells with integrated HIV provirus remain transcriptionally active in the lung during HAART [45]. Nonetheless, our results suggest that gp120 may play an important role in airway mucus production in HIV-infected patients and SIV-infected macaques, and excessive mucus production could contribute to HIV-related obstructive lung diseases. Because HIV gp120 uses the α7-nAChR/GABA_A_Rα2 pathway to trigger airway mucus formation and mucous cell hyperplasia, α7nAChR and GABA_A_Rα2 antagonists may have beneficial effects in reducing mucus formation and development of obstructive lung diseases and lung infections in HIV-infected subjects.
